# Highlights from the 2013 National Cancer Research Institute Conference

**DOI:** 10.3332/ecancer.2014.386

**Published:** 2014-01-14

**Authors:** Ian Lewis

**Affiliations:** Department of Research, Tenovus, Cardiff CF14 5BD, UK

**Keywords:** conference highlights, National Cancer Research Institute, screening, inflammation, immunotherapy, DNA repair, palliative care, predisposition genes, patient/public involvement, model system

## Abstract

Cancer research is a multifaceted endeavour that incorporates not only a myriad of techniques and specialties but also encompasses a huge range of disease types. The National Cancer Research Institute (NCRI) is a UK partnership comprising 21 charity and government funders of cancer research along with the Association of British Pharmaceutical Industry. Each year, the NCRI hosts the largest cancer meeting in the UK; bringing together members of the UK cancer research community, research leaders from around the world, health professionals, service users, research funders, and industry to discuss the latest findings in cancer research from a wide range of disciplines. The 2013 NCRI Conference attracted over 1700 delegates and 150 speakers from 15 different countries. The conference programme covered a large range of topic areas including prevention, screening, model systems, the provision of information, survivorship, and end-of-life care. This conference report gives an overview of the plenary sessions at the conference as well as highlights from the parallel sessions.

## Introduction

In the ten years leading up to 2011, spending on cancer research in the UK has increased by 62%, with over £521 million a year currently invested in peer-reviewed research [1]. However, despite the rapid expansion in cancer research spending, this investment is still dwarfed by the economic burden of cancer in the UK and across the European Union’s 27 countries, which has been estimated to have reached €126 billion per year in 2009 [2]. With an expanding and ageing population [3], this burden is set to increase.

The National Cancer Research Institute (NCRI) is a partnership comprising 21 charity and government funders of cancer research based within the UK together with the Association of British Pharmaceutical Industry. The primary aim of the NCRI is to coordinate research and funding strategies for the ultimate benefit of patients, carers, and all people affected by cancer.

Each year, the NCRI Conference, the largest meeting of its kind in the UK, brings together some of the very best researchers in the world from a wide range of disciplines to update on their latest findings, debate the current hot topics in cancer research, and explore multidisciplinary partnerships. One of the great strengths of the NCRI Conference is that this knowledge sharing is very much underpinned by a strong emphasis on patient public involvement and engagement at all levels.

The ninth annual NCRI Conference, held in Liverpool from 3–5 November 2013, attracted over 1700 delegates, with 150 speakers from 15 different countries. This year, the conference programme consisted of plenary lectures, symposia, workshops, proffered paper sessions, clinical trial showcases, and parallel sessions. The parallel sessions were grouped into the following themes: (a) diagnosis and therapy; (b) epidemiology and prevention; (c) health services research; (d) information, patients, and the public; (e) survivorship and end-of-life care; (f) the cancer cell and model systems; and (g) tumour specific research.

This paper outlines some of the highlights from the conference and seeks to reflect the diversity of the research discussed.

## Plenary lectures

### Opening lecture

If there was not enough of a challenge involved in gathering and marshalling the very best and brightest in cancer research to Liverpool for this year’s conference, Chair of the Scientific Committee Prof Gerard Evan from Cambridge was also called upon as a last-minute opening speaker, as Prof Neal Rosen from Memorial Sloan-Kettering Cancer Center New York could not attend due to family commitments.

Evan’s talk proved to be an excellent way to frame the conference with the key questions: ‘why is cancer so difficult to treat and what properties make a molecular target a candidate for cancer therapy’? He went on to describe one such potential target, Myc, a transcription factor with broad influence that acts as an ‘accelerator’ gene in many tumour types. He has shown in tumour-bearing mouse models that genetic knockdown of Myc expression had a dramatic effect on tumour size, with mild and reversible side effects. Whilst these effects were temporary and the development of drugs based on this principle of Myc inhibition remain a challenge, Prof Evan thinks that this can be overcome. For a target that is such a critical driver of cell proliferation, the promise of a generic cancer treatment is an extremely inviting one and could signal a shift from an era of ‘personalised’ medicine to an era of ‘impersonalised’ medicine.

### Cervical cancer, Human papillomavirus, and screening

With a cervical screening programme and a widespread human papillomavirus (HPV) vaccination programme now in place in the UK, the question ‘cervical cancer—problem solved?’ posed by Prof Peter Sasieni from the Wolfson Institute of Preventative Medicine, London, drew much attention from the delegates at the conference and also from the wider UK press. In his talk, he outlined the advantages and limitations of conventional cervical screening, HPV testing, and HPV vaccination in the control of cervical cancer. Whilst vaccination could almost eradicate the disease in the developed world, this is ‘today’s solution for tomorrow’s problem’ and points out that women born before 1990 will not be protected and will require screening for many years to come. However, on this point, Sasieni highlights that the benefits of screening decrease with age but it is difficult to know at what age to stop. Conversely, the evidence shows that the benefit of screening women in their early 20s does not outweigh the risk of overtreatment.

Sasieni also points of that these arguments will not apply to women in the third world, who account for a large proportion of the global burden of cervical cancer, where the vaccine is unaffordable. He highlighted that this picture may not be so clear cut in the UK either. A related poster presentation by Dr Jo Waller and his colleagues at University College, London, provided data to show that girls from some ethnic groups in the UK were less likely to be vaccinated than others and would be less likely to attend screening for cervical cancer when invited as adults. This suggests a need to better understand the reasons for ethnic inequalities in uptake to ensure that more unvaccinated women understand the importance of cervical screening.

### BRAF and RAS signalling in melanoma

The protein kinase BRAF is mutated in about half of all melanomas. Prof Richard Marais outlined in his talk how his work on BRAF underpinned the development of the drug vemurafenib, a small molecule kinase inhibitor, which provides a short period of remission followed by the development of resistance. As with most of the cancers, the development of resistance can be complicated and multifaceted. However, in his talk, Marais focused on how epidermal growth factor receptor (EGFR) up regulation and mutations in other kinases can drive resistance to B-Raf inhibitors in tumours with this mutation. Marais is now developing fully characterised tumours derived from melanoma patient xenographs that have been treated with targeted agents. This platform will then be used to predict resistance mechanisms allowing for the pre-emptive treatment of resistance before it has the opportunity to arise.

### Chromosomal instability

Prof Charles Swanton of the University College London Cancer Institute presented a talk titled *Chromosomal Instability and Hopeful Monsters*. The ‘hopeful monsters’ in question are a reference to the work of evolutionary biologists Richard Goldschmidt and Stephen J. Gould, which theorises that evolutionary transformations have occurred in large leaps between species due to macromutations. Swanton outlines how chromosomal instability can be considered a macromutational event capable of producing large phenotypic leaps that drive the rapid evolution of tumours. He also discussed the process of genome doubling, where cancer cells end up with twice the number of chromosomes as they should, allowing the cells to survive, even in the presence of major genetic problems. Both genome instability and doubling are associated with a poor prognosis. In some cases, treatments that seek to address this instability can be more effective than agents that seek to compound it.

### Inflammation and cancer

Whilst it is well known that tumours can be highly heterogeneous, Prof Lisa M Coussens from the Knight Cancer Institute, Portland, described how only around half of all the cells that make up a solid tumour are actually cancer cells with the remainder comprising of normal cells that make up the tumour microenvironment.

A large proportion of these are leukocytes that, rather than protecting us from harm, are instead driving protumourigenic pathways. By studying transgenic mouse models of skin, lung, breast, and pancreas cancer development, Prof Coussens and her team have shown that adaptive leukocytes differentially regulate myeloid recruitment by organ-dependent mechanisms and that these myeloid cells can prevent CD8+ T cells from killing the tumour in maintaining a microenvironment that allows the tumour to continue to thrive and spread.

By blocking this myeloid response in these preclinical mouse models, they have been able to slow the growth of the tumour, improve the effectiveness of chemotherapy, and significantly reduce the spread of the tumour. This approach is now being investigated in clinical trials including a phase Ia/II trial of the tyrosine kinase inhibitor PLX3357 in combination with eribulin for the treatment of metastatic breast cancer.

### CRUK Lifetime Achievement Award

One of the annual highlights of the NCRI Conference is the Cancer Research UK Lifetime Achievement Award lecture, and this year was no exception. Prof Sir Bruce Ponder, Director of the CRUK Cambridge Institute, gave a wonderful presentation full of inspirational advice, particularly to early career researchers, and served as an example of how great storytellers and orators bring research to life.

As well as reflecting on his career to date, Prof Ponder spoke about his research regarding polygenic susceptibility to cancer rather than individual genes with strong effects, and how these networks of genes could help us to identify individuals at a high risk and target them for earlier intervention.

### DNA repair pathways

Our DNA is constantly subjected to damage from endogenous sources such as reactive oxygen species produced as byproducts of oxidative metabolism, from the breakdown of replication forks during cell growth, or by the agents in the environment such as ionising radiation or carcinogenic chemicals. Therefore, DNA repair pathways are vital in safeguarding the integrity of our DNA. In his plenary lecture, Prof Stephen West from the Cancer Research UK London Research Institute focused on a double-strand break, one particularly dangerous form of damage than can occur during DNA replication. When these occur, they need to be repaired to avoid DNA translocation or partial chromosome loss. West highlighted some of the known tumour suppressors that are involved in this repair mechanism including PALB2, BLM, and BRCA2. West and his group have purified these proteins and shown how mutations in these genes can give rise to breast cancer, Bloom’s syndrome, and Fanconi anaemia, respectively.

### Translational science in nonsmall cell lung cancer

At last year’s conference, Dr Shepherd gave a talk on the search for the ‘holy grail’ of lung cancer, a molecular test that could accurately predict which patients would respond to certain targeted therapies and how likely this was to improve their outcomes. However, as the subtitle to her talk this year stated, ‘It’s not quite as easy as we once thought’. Dr Shepherd focused mainly on two select examples that are frequently mutated in nonsmall cell lung cancer (NSCLC).

Despite being frequently mutated in NSCLC, several large studies have failed to link KRAS to differential benefit of chemotherapy. The only exception to this is in a subgroup of patients with a mutation at Codon-13, but the numbers identified in this cohort are currently too small to achieve a statistically significant link. Additionally, whilst mutations in Exons 19 and 21 have been found to predict a very strong response to EGFR tyrosine-kinase inhibitors, all patients in this group still relapse and succumb to their disease.

The identification of mutations created by the abnormal fusion of echinoderm microtubule-associated protein-like 4 (EML4) gene and the anaplastic lymphoma kinase (ALK) gene has led to the rapid approval of drugs that target the protein produced EML4 ALK. Whilst this protein appears to be a promising candidate for targeting in NSCLCs where it promotes and maintains the malignant behaviour of cancer cells, resistance is already reported to have occurred. However, second generation inhibitors are now being developed with options for combination therapy also being explored.

### Palliative medicine and end-of-life care

With an ageing population and an ever increasing incidence of cancer, the need for effective palliative care services has never been more important. In his plenary lecture *Improving Value in Cancer Care: The Case for Palliative Medicine*, Sean Morrison of the Hertzberg Palliative Care Institute in New York reviewed the evidence examining the effect of palliative care on patients’ quality of life, patient and family satisfaction, and healthcare expenditures. He included data from a recently completed multisite study carried out in the United States that looked at the effect of palliative care on cancer care which showed that those who were given palliative care had a better quality of life, lived longer, and that the average cost of their care was less. With these demonstrable outcomes in mind, he suggested that we need to change the way in which we consider palliative care as simply something we undertake at end of life but rather as a holistic package of care designed to support all patients and carers alongside potentially curative treatment regimes.

### Cancer predisposition genes

The final plenary lecture at the conference was delivered by Prof Nazneen Rahman of the Institute of Cancer Research, London. Her talk focused on cancer predisposition genes, genes in which rare germline mutations result in clinically important increased risk of developing cancer. Over the last 30 years, more than 100 such genes have been identified, which can predict an increased risk of over 40 different cancers.

Faster and cheaper sequencing technologies are leading to a new wave of cancer predisposition genes being discovered. Prof Rahman spoke about the UK Breast and Ovarian Susceptibility (BOCS) Study, which with 12,625 samples from individuals who have either been diagnosed with breast cancer, ovarian cancer, or tested positive for BRCA1/2 mutations, is the biggest study of its kind in the world. The purpose of this study is to identify the genetic factors involved in causing breast and/or ovarian cancer.

This study has already identified several genes and genetic variants that predispose to these cancers. For example, the BOCS study has shown that the gain of function mutations in the DNA repair gene PPM1D is associated with an increased risk of breast and ovarian cancer.

Whilst there are many potential clinical benefits to identifying these genes, Prof Rahman also gave a note of caution that there are potential pitfalls and the risk that they might actually lead to harm for some individuals.

## Parallel sessions

### Diagnosis and therapy

The session *Smarter Surgery for Better Outcomes *chaired by Dion Morton of the University of Birmingham in the UK brought an audience of researchers from a range of disciplines to look at the connections that can be made between surgery and a range of other research and treatment areas. These include the opportunities that surgery presents for biobanking, the role oncologists can play in helping to reduce the extent and consequences of a surgical procedure and the efficient design, and delivery of randomised control trials in surgical oncology.

As our understanding of the epigenome increases, it throws up exciting opportunities for novel anticancer approaches. The session *Targeting the Epigenome *covered some of the targets that have been identified to date, how these are being turned into potentially effective treatments, and how academia and industry are working together to create a critical mass of infrastructure and expertise to drive this research.

Another exciting area of research currently being applied to oncology is nanotechnology. In the parallel session titled *The Application of Nanotechnology*, many areas where nanotechnology is now being utilised in oncology were highlighted including drug delivery and development, early diagnosis, and imaging.

An area of research that is much more established is immunology and immunotherapy. There were several talks that focused on this area including how the analysis of T-cell responses combined with exome sequencing can be used to uncover T-cell reactivity against mutated proteins, the identification of novel targets for T-cell therapy on solid tumours, immunological therapies that target paediatric leukaemia and neuroblastoma, and the use of model systems to test IgE-based immunotherapy.

### Information, patients, and the public

One of the great strengths of the NCRI is its involvement of patients and the public and its emphasis on the importance of the wider dissemination of cancer research. This is reflected by one of the key themes of the conference being on information, patients, and the public. The sessions that came under this theme covered a broad range of subjects and highlighted the importance and impact that research in these areas can have on people affected by cancer, particularly, in terms of survivorship and end-of-life care.

Five-year survival rates from childhood cancer have increased from under 30% in the 1960s to over 80% today. As a result of this, there are an ever-growing number of childhood cancer survivors who have reached adulthood. In the session titled *Life Altering Late Effects and Their Management in Survivors of Childhood Malignancy*, the speakers looked at issues such as the frequency and timing of late mortality and the consequences of treatment with regards to their long-term physical and psychosocial health and fertility issues.

The patient advocacy group Independent Patients Voice ran a session called *The Issues with Tissues *encouraged patients and professionals to come together to discuss current and potential opportunities for research involving tissue samples as well as potential barriers to collection and use by the researchers.

Chronic daily breathlessness is highly prevalent amongst cancer patients, with around 70% of all terminal cancer patients suffering breathlessness in their last six weeks. The session *Refractory Breathlessness: Mechanisms and Management *looked at the multifactorial causes of this condition and how increasing our knowledge of these factors can lead to more effective management through medication and successful strategies for self-management.

Clinical depression is common in individuals with advanced disease, with its prevalence increasing towards end of life. It not only has an adverse effect on the quality of life of people with advanced cancer but is also an independent predictor of mortality. In the session *Depression in Advanced Cancer—The Orphan Symptom?, *chaired by Prof Mari Lloyd-Williams of the University of Liverpool, the speakers gave fascinating insights into the identification of depression, advances in its treatment, and the role that spirituality plays in the provision of end-of-life care for cancer patients.

Despite the fact that carers have an essential role in the provision of support at all phases of the cancer journey, relatively little research has been carried out to develop interventions that support care givers or the effectiveness of the interventions that currently exist. With an ageing population, there will be an increase in the number of people requiring care with a concomitant decrease in the pool of people able to provide that care. In the session *Supporting Family Carers: International Evidence*, a range of talks reflected on the support needed by carers of cancer patients in the UK, Scandinavia, and Canada as well as comparing the differences between urban and rural care at the end of life and its implications for policy and service development.

### Epidemiology and prevention

A big thank you to Dr Anna Gavin of the Northern Ireland Cancer Registry for possibly the most interesting fact of the conference. In the session *Real World Data: Opportunities and Challenges, *she pointed out that each day there is enough data produced to fill 150,000 iPads. With regards to cancer, there are at least 500 population-based cancer registries around the world. Whilst the collection of this data can be expensive, the linkage of routine cancer patient data and the services they access has improved the knowledge available to service providers, researchers, and patients themselves.

If the session on real-world data had the most interesting fact, the session titled *The End Game for Tobacco Control in the UK: Key priorities for Science and Policy to Reduce Smoking *had the most shocking. In the 21st century, approximately one billion people will die due to tobacco use. This session compared the UK with other countries and looked at how we prevent people from taking up smoking in the first instance, several potential ‘end game’ solutions to reduce the use of tobacco products, and how the tobacco inductry is pushing back against these initiatives.

### The cancer cell and model systems

The session *The Importance of Knowing Which Way Is Up *looked at how polar cell organisation influences cell growth and death in the context of an epithelium. The loss of polarity in epithelial cells is associated with cancer progression with increasing evidence that protein involved in polarity can be potent suppressors of invasion. In this session, we learned about the importance of molecules called fat cadherins, which are essential for the orientation of our cells, and how these molecules are essential for the regulation of mitochondrial activity.

We also saw how the identification of genes involved in the normal development of epithelial organs can be subverted to create cancer-like structures and how errors in cell division can create too many cancer stem cells leading to carcinogenesis. A greater understanding of this emerging area is a potential source of exciting new targets for the treatment of epithelial cancers.

The migration of cancer cells into the surrounding tissue is an early event in metastasis. What the session *Cell Migration in Tumours *showed was the range of strategies that cancer cells can employ to move and how quickly they can shift between these different strategies.

Among the less obvious differences between mouse and man are how our immune systems function and how we metabolise drugs. However, these differences are acutely important in the context of using mouse models to investigate drug treatments. In the session *Humanising mouse models of cancer: How relevant are current models?*, a number of genetically modified mouse models of cancer were discussed including models of pancreatic cancer that contain predisposing gene mutations or patient-derived xenographs and also mouse models of common childhood malignancies that share common disease-associated oncogenes and genomic alterations.

### Tumour-specific research

As well as research areas that underpin the wider biological processes of cancer, there are also a number of tumour specific research areas that are highlighted. This year, there were sessions on the stratification and clinical management of adult high-grade glioma, emerging molecular insights regarding neuroendocrine tumours, a review of the latest developments in sarcoma research, and a session looking at the impact of the latest translational research on the clinical management of breast cancer.

## Conclusions

This year’s NCRI Conference covered a large range of topics, reflecting the strength and diversity of the cancer research community in the UK and also the ability of the conference to attract high-profile speakers from around the world. Next year, the NCRI Conference enters its tenth year, which provides a perfect opportunity for the conference to not only look forward to the latest advances in cancer treatment, information, support, and care but also reflect on a decade where much has been achieved in cancer research, with much more left to uncover.
